# Humanized Culture Medium for Clinical-Grade Generation of Erythroid Cells from Umbilical Cord Blood CD34^+^ Cells

**DOI:** 10.34172/apb.2021.031

**Published:** 2020-02-29

**Authors:** Majid Zamani, Yoda Yaghoubi, Adel Naimi, Ali Hassanzadeh, Ramin Pourakbari, Leili Aghebati-Maleki, Roza Motavalli, Afsoon Aghlmandi, Amir Mehdizadeh, Mehdi Nazari, Mehdi Yousefi, Ali Akbar Movassaghpour

**Affiliations:** ^1^Department of Medical Laboratory Sciences, Faculty of Allied Medicine, Gonabad University of Medical Sciences, Gonabad, Iran.; ^2^Stem Cell Research Center, Tabriz University of Medical Sciences, Tabriz, Iran.; ^3^Cellular and Molecular Research Center, Sabzevar University of Medical Sciences, Sabzevar, Iran.; ^4^Department of Tissue Engineering and Applied Cell Sciences, Tehran University of Medical Sciences, Tehran, Iran.; ^5^Immunology Research Center, Tabriz University of Medical Sciences, Tabriz, Iran.; ^6^Endocrine Research Center, Tabriz University of Medical Sciences, Tabriz, Iran.; ^7^Department of Anesthesiology, Faculty of Medicine, Tabriz University of Medical Sciences, Tabriz, Iran.; ^8^Hematology and Oncology Research Center, Tabriz University of Medical Sciences, Tabriz, Iran.

**Keywords:** Human platelet lysate, Fetal bovine serum, CD34^+^ hematopoietic stem cells, Erythroid differentiation

## Abstract

***Purpose:*** Transfusion of red blood cells (RBCs) is a supportive and common treatment in surgical care, trauma, and anemia. However, in vivo production of RBC seems to be a suitable alternative for blood transfusions due to the limitation of blood resources, the possibility of disease transmission, immune reactions, and the presence of rare blood groups. Cell cultures require serum-free or culture media supplemented with highly expensive animal serum, which can transmit xenoviruses. Platelet lysate (PL) can be considered as a suitable alternative containing a high level of growth factors and a low production cost.

***Methods:*** Three-step culture media supplemented with PL or fetal bovine serum (FBS) were used for proliferation and differentiation of CD34^+^ umbilical cord blood stem cells to erythrocytes in co-culture with bone marrow mesenchymal stem cells (BM-MSCs). The cells were cultivated for 15 days and cell proliferation and expansion were assessed using cell counts at different days. Erythroid differentiation genes, CD71 and glycophorin A expression levels were evaluated.

***Results:*** Maximum hematopoietic stem cells (HSCs) proliferation was observed on day 15 in PL-containing medium (99±17×10^3^-fold). Gene expression and surface markers showed higher differentiation of cells in PL-containing medium.

***Conclusion:*** The results of this study indicate that PL can enhance erythroid proliferation and differentiation of CD34^+^ HSCs. PL can also be used as a proper alternative for FBS in the culture medium and HSCs differentiation.

## Introduction

Cell culture is performed in a serum-free medium or within animal/human serum in supplemented media.^1^ Animal serum, especially fetal bovine serum (FBS), is the standard growth supplement for cell culture. Nevertheless, the use of FBS has disadvantages such as high cost, transmission risk of prions and pathogens, xenogeneic immune reactions, batch-to-batch variation, and ethical issues for animal welfare.^2^

In recent years, a number of alternatives with human origin such as human serum, platelet derivatives, umbilical cord blood serum, autologous and allogeneic serum albumin have been introduced as substitutes for animal serum.^2^

Platelet lysate (PL) is a blood product and a suitable alternative for FBS containing growth factors and chemokine (CXC) such as basic fibroblast-derived growth factor (b-FGF), insulin like growth factor-I (IGF-I), epidermal growth factor (EGF), platelet-derived growth factor (PDGF), hepatocyte growth factor (HGF), connective tissue growth factor (CTGF), granulocyte-colony stimulating factor (G-CSF), granulocyte-macrophage colony-stimulating factor (GM-CSF), C­XC ligand 1, CXCL2, CXCL3, CXCL4 (PF4), CXCL5, CXCL10, and CXCL12 (SDF-1), which can promote cell growth, proliferation and differentiation in comparison with FBS.^3,4^

Red blood cells (RBCs) are continuously produced in the bone marrow (BM) from hematopoietic stem cells (HSCs), which constitute about 0.01% of nucleated cells in BM.^5,6^ HSCs are also able to differentiate into blood cells and have the self-renewal capacity to maintain HSC pool in BM.^5,6^ HSCs are in a specific condition in BM with a great impact on hematopoiesis, differentiation and maintenance of different cells in BM. Additionally, BM stromal cells such as fibroblasts, reticular cells, endothelial cells, mesenchymal stem cells (MSCs), adipocytes, and osteoblasts interact with HSCs.^5,7^

RBCs transfusion is a supportive care in trauma, surgery, anemia, and therapy for hematological malignancies.^1^ Nearly 90 million units of blood are annually donated worldwide.^8^ However, transfusion of blood is faced with limitations due to short life-spans, blood deficiency for rare blood groups, immunoreactivity potential , and the transmission of viruses and diseases.^9^ *In vitro*-generated RBCs are an appropriate alternative to these blood units and can be used for drug delivery and discovery, as well as reagent RBCs for antibody identification and blood transfusion.^1^

In this study, we aimed to investigate the effect of PL replacement with FBS on the pattern of erythroid differentiation and RBCs generation for blood transfusion, delivery and discovery of drugs, and reagent RBCs for antibody identification.

## Materials and Methods

### PL preparation

PL was prepared from expired platelet bags from Iranian Blood Transfusion Organization by freeze/thaw protocol. For this purpose, 25 platelet bags were collected and pooled in five batches and the platelet suspension was frozen at -80°C for 24 hours. Subsequently, the suspension thawed at 37°C for 15 minutes to release PDGFs, and this cycle was repeated three times. Then, PL was centrifuged at 1600 g for 20 min at 4°C to remove platelet fragments and leukocytes, the supernatant was filtered by 0.2 μm filter and the level of growth factors in it was evaluated. Finally, all the prepared PLs were combined and stored at -80°C for future use.

### Growth factor immunoassays

The concentration of growth factors in PL was assessed in platelet batches. For this purpose, after PL preparation, the PL growth factors, including transforming growth factor beta (TGF-β), IGF-1, EGF, PDGF-AB, bFGF, and vascular endothelial growth factor (VEGF), were evaluated by ELISA (enzyme-linked immunosorbent assay, all from R&D, USA).

### MSCs culture

Bone marrow mesenchymal stem cells (BM-MSCs) were purchased (Gene O Cell, Iran) and cultured in DMEM medium (Dulbecco Modified Eagle Medium, Sigma, USA) containing 10% PL or FBS (Gibco, USA), 1% glutamine (Invitrogen, USA) and 10 IU/mL penicillin/streptomycin (Gibco, USA) and seeded at 2×10^5^ cells/mL within T-25 culture flasks. The cultures were incubated at 37°C and 5% CO_2_ and the medium was exchanged twice a week until the cultures reached 80–90% confluence. The cells were passaged twice, detached using trypsin/EDTA (Gibco, USA) and were subject to evaluation of population doubling time (PDT) and growth curves.

### PDT calculation 

To determine the PDT, passage #5 of the cells was plated at 10^4^ cells/cm^2^ in T-25 culture flasks for a while, When the cells were approximately 80% confluent (approximately 8 and 7 days in FBS- and PL-containing media, respectively), all the cultures were terminated by trypsinization to determine the cell number. The following equations were used to determine PDT and PDN:

PDT= culture time (CT)/ population doubling number (PDN)

PDN= log N/N_0_×3.31 (N stands for the final number of cells and N_0_ is the initial number of the cells).

### Evaluation of growth curve 

To determine BM-MSCs growth curve, the cells from passage #5 in the culture medium supplemented with PL or FBS were plated at 5×10^4^ cells/well in 6-well culture plates and left to become confluent. A number of wells were trypsinized every other day and the cells were counted using a hemocytometer. Then, the growth curves were plotted by considering the obtained data.

### Isolation of UCB CD34^+^ cells 

CB was collected at Al-Zahra Hospital affiliated to Tabriz University of Medical Sciences from full-term healthy deliveries after obtaining informed consent from mothers aged 25-35 years with no abnormal laboratory findings. Mononuclear cells (MNCs) fraction was separated within 8 hours after delivery and MNCs were enriched by Ficoll/Histopaque (density: 1.077 g/mL). CD34^+^ cells were isolated by positive selection using immuno-magnetic microbeads and MiniMACS columns (Miltenyi Biotech, USA).

### Culture of UCB CD34^+^ cells 

Purified UCB CD34^+^ cells were cultured in a three-step expansion as described earlier with minor modifications.^10^ Briefly, the cells were cultured in an erythroid differentiation medium consisting of Iscove’s modified Dulbecco’s medium (IMDM, Gibco, USA) supplemented with 10% PL or FBS, 4 mmol/L stabilized glutamine, 220 µg/mL iron-saturated human transferrin, 10 µg/mL ferrous sulfate, 100 ng/mL ferric nitrate, and 20 µg/mL insulin (all from Sigma, USA). The expansion procedure was done in three steps. In the first step (day 0-7), 10^4^/mL CD34^+^ cells were cultured in IMDM in the presence of 10-^6^ M hydrocortisone (Sigma, USA), 100 ng/mL SCF (stem cell factor, R&D Systems, UK), 5 ng/mL IL-3 (Amgen Biologicals, USA), and 3 IU/mL EPO (Erythropoietin, Boehringer, USA). Fresh medium containing SCF, IL-3, EPO, and hydrocortisone was used for dilution of one volume of cell culture in four volumes of fresh medium on day 4. In the second step (day 7-10), the cells were co-cultured with BM-MSCs at 10^5^/mL in IMDM supplemented with EPO. In the third step (day 10-15), the cells were co-cultured with BM-MSCs in IMDM without cytokine. All the cells were incubated at 37°C and 5% CO^2^. The cells cultured in FBS-containing medium were considered as the control group.

### Immunophenotypic analysis

The differentiation of CD34^+^ cells was investigated by flow cytometric analysis (BD FACS Calibour, USA). The cells were labeled with anti-CD71-APC and anti-glycophorin A-PE (BD Biosciences, USA) (1/10) using 1% BSA (w/v) in PBS for 20 minutes at 4°C on days 0, 7 and 15 of culture. The cells were analyzed using 2-color flow cytometry by combining PE-labelled and APC-labelled antibodies and the analysis was implemented on a BD FACS Calibour flow cytometer (BD Biosciences, USA) and FlowJo X10 (Tree Star Inc., USA) software.

### RNA isolation and quantification 

To investigate the effect of PL on erythroid differentiation, we studied the expression of erythroid-specific genes, including transcription factor *GATA-1*, *GATA-2*, *NFE2*, g and b globins. For this purpose, at day 8, the cells were harvested for real-time polymerase chain reaction (PCR) analysis. Gene expression was estimated as fold expression relative to the corresponding gene expression at cells cultivated in FBS-containing medium. Total RNA was extracted by RNA Isolation Kit (Yekta Tajhiz Azma, Iran).

Complementary DNA (cDNA) was then synthesized by Transcriptor cDNA Synthesis Kit (Takarabio, japan). Quantitative real-time PCR (qPCR) was performed using Light Cycler Fast Start DNA Master SYBR Green (Amplicon, Denmark) and gene-specific primers (Sinaclon, Iran). The nucleotide sequence of primers is listed in Table 1. SYBR Green fluorescence was used to monitor product accumulation. qPCR conditions for *GAPDH*, *GATA-1*, *GATA2*, *NFE2*, g and b globin target genes were as follows. Pre-incubation at 95°C for 10 minutes and 40 amplification cycles: 5 seconds at 95°C for denaturation, 10 sec at 60°C for annealing, and 10 seconds at 72°C for extension. Specificity of each PCR product was checked by melting curve analysis. LightCycler software was employed for PCR efficiency and exponential amplification was calculated from corresponding standard curve slopes. For normalization of results, human GAPDH was used as the reference gene.

**Table 1:** Primer sequences for Real-time polymerase chain reaction.

Gene	Primer Sequence
GATA1	F: CACGACACTGTGGCGGAGAAAT
	R: TTCCAGATGCCTTGCGGTTTCG
GATA2	F: CTGTTCAGAAGGCCGGGAG
	R: TTCGCTTGGGCTTGATGAGT
NFE2	F: GATCCTCGTCCAGCAGTGTC
	R: TGGCTCTAGAAACCTGTGGTG
Globin β	F: GCACGTGGATCCTGAGAACT
	R: ATGGGCCAGCACACAGAC
Globin γ	F: ATGCCATAAAGCACCTGGAT
	R: AAACGGTCACCAGCACATTT
GAPDH	F: ACCCATCACCATCTTCCAGGAG
	R: GAAGGGGCGGAGATGATGAC

The following equation was used to determine relative gene expression (RGE):

RGE = (amplification target gene)^ΔCttarget(control-sample)^/ (amplification GAPDH)^ΔCtGAPDH(control-sample)^

The day 8 erythroid cells cultivated in FBS-containing medium were the control in the above equation and the sample was day 8 erythroid cells cultured in PL-containing medium. Δ^Ct^ represents the difference in cycle threshold (Ct).

### Statistical analysis

Data normality was analyzed with column statistic GraphPad Prism (version 8.0.2) and* P* < 00.05 was considered as statistically significant. The results were presented as mean± standard deviation (SD).

## Results

### PL growth factors 

**Figure 1:**
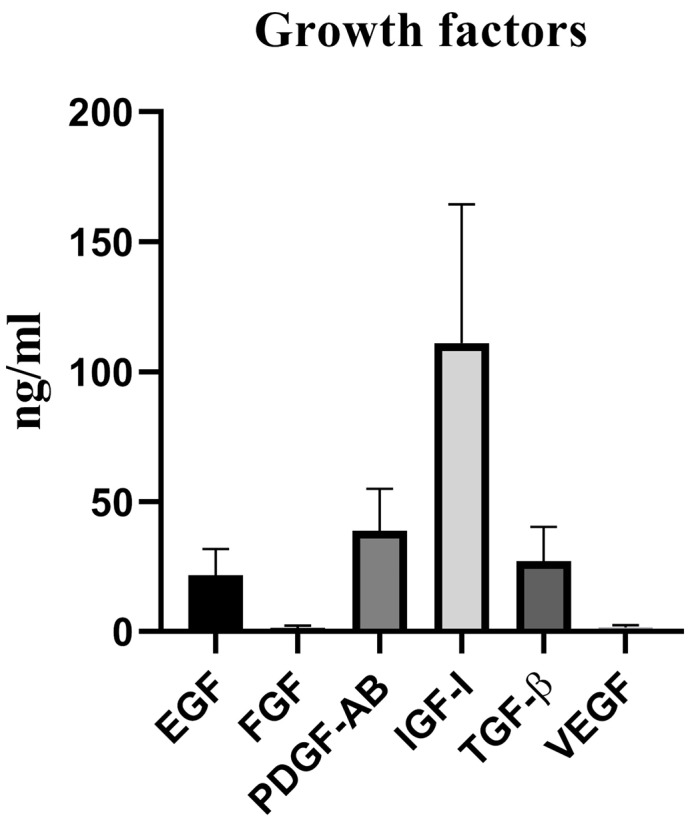
The quantity of five pooled bags PL growth factors. Data are presented as Mean± standard deviation. PL: platelet lysate; EGF: epidermal growth factor; b-FGF: basic fibroblast growth factor; PDGF: platelet-derived growth factor; IGF-I: insulin like growth factor-I; VEGF: vascular endothelial growth factor; TGFβ: transforming growth factor.

The concentration of main growth factors was determined in five pooled platelet bags. The mean concentrations of human TGF-β, IGF-I, EGF, PDGF-AB, b-FGF and VEGF were 27.18 ± 13.19 ng/mL, 111.1 ± 53.37 ng/mL, 21.54 ± 10.34 ng/mL, 38.84 ± 16.29 ng/mL, 1.36 ± 0.88 ng/mL and VEGF 1.58 ± 0.85 ng/mL in all PLs batch, respectively (Figure 1).

### MSCs culture

**Figure 2:**
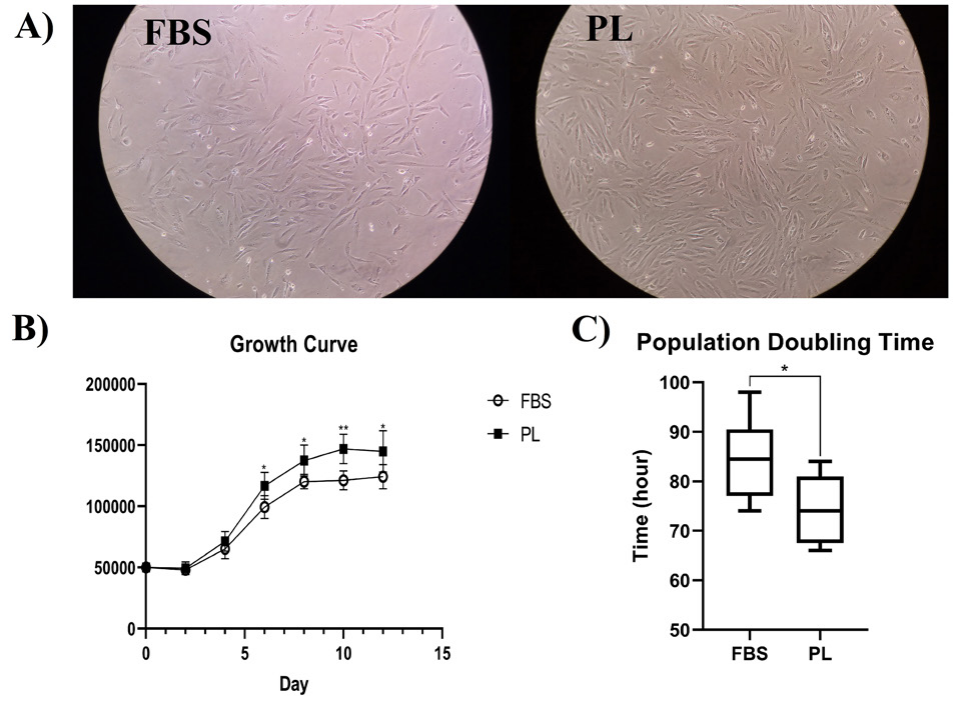
MSCs culture outcomes in FBS- or PL-containing media. (**A)** Morphology of MSCs cultivated in two different media (magnification ×40). (**B)** The growth curve of MSCs. (**C)** The population doubling time of MSCs. Data are presented as mean± standard deviation of six independent experiments (*P < 00.05, ** P <0.01).

The growth-stimulating activity of PL for BM-MSCs was compared to that of DMEM supplemented with 10% FBS as the standard medium. The cultured cells showed no obvious differences of morphology in both media. The cells also attached to plastic flasks and showed small spindle-like shapes with a few small transparent cells among them (Figure 2A).

### Growth curve

The growth curves of cells cultivated in PL- or FBS-containing media revealed the same ‘exponential’ growth pattern with three lag, log, and plateau phases. However, the growth rate of BM-MSCs cultured in PL-containing media was significantly faster than that of BM-MSCs cultured in FBS-containing media (*P* < 00.05) (Figure 2B).

### Population doubling time 

The growth kinetics of cells cultured in PL- or FBS-containing media was compared using the PDT. The mean PDT of BM-MSCs cultured in PL- and FBS-containing media was 74.33±6.89 and 84.5±8.47 hours, respectively (Figure 2C). The BM-MSCs cultured in PL-containing media also showed significantly faster growth kinetics than that of BM-MSCs cultured in FBS- containing media (*P* < 00.05).

### Isolation of UCB CD34^+^ cells 

**Figure 3:**
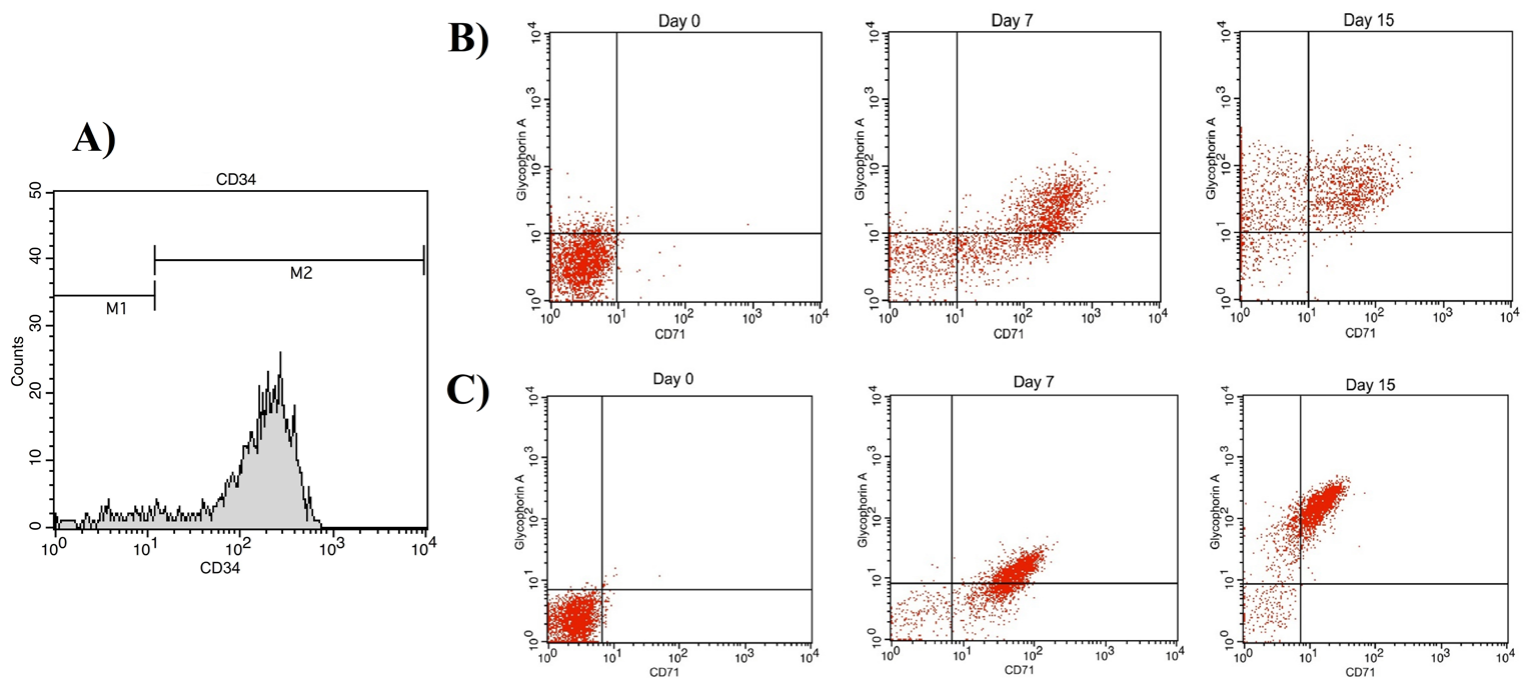
The result of immunophenotype assay. (**A)** Purity of isolated CD34^+^ cells. For erythroid differentiation assay, CD71 and Glycophorin A expressions were investigated at day 0, 7 and 15. (**B)** CD71 and Glycophorin A expressions in cells cultivated in FBS-containing media. (**C)** CD71 and Glycophorin A expressions in cells cultivated in PL-containing media. Gates for cells stained with Glycophorin A-PE and CD71-APC define positivity of cells for these surface markers i.e. lower left quadrant: CD71-Glycophorin A-; upper left quadrant: CD71- Glycophorin A^+^; upper right quadrant: CD71^+^ Glycophorin A^+^; and lower right quadrant: CD71^+^ Glycophorin A-.

60±10 mL UCB sample was collected for CD34^+^ isolation. The mean CD34^+^ cell purity was 94.79% as assessed by flow cytometry (Figure 3A) (FACSCalibur, Becton Dickinson) and cell viability was higher than 95% as determined using trypan blue dye.

### Expansion of erythroid cells 

**Figure 4:**
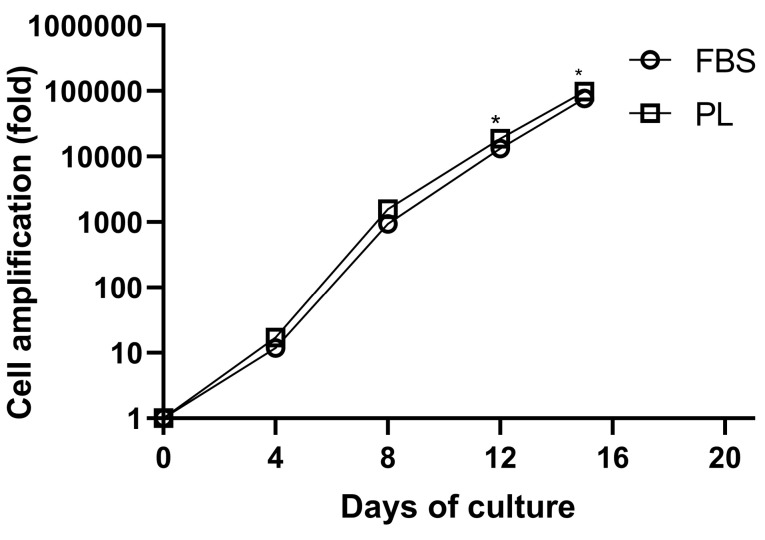
Cultivated cord blood CD34^+^ HSCs according to the three-step stimulation protocol in medium containing PL or FBS. Data are obtained from six independent experiments (*P < 0 0.05).

We used a three-step protocol for culture of HSCs and their differentiation into the erythroid lineage. Firstly, erythroid differentiation and proliferation were induced in IMDM supplemented with 10% PL or FBS, SCF, IL-3, and EPO for 7 day. Secondly, the cells were co-cultured with additional erythropoietin on human BM-MSCs for 3 day. In the third step, the cells were incubated on BM-MSCs without cytokine for 5 days. By day 15, the mean cell amplification fold of CD34^+^ cells reached a plateau of 99±17×10^3^ and 77±16×10^3^ fold in PL- and FBS-containing media, respectively, and the difference between expansion of the two groups was statistically significant (*P* < 0.05) (Figure 4).

### Immunophenotypical changes of erythroid cells

**Figure 5:**
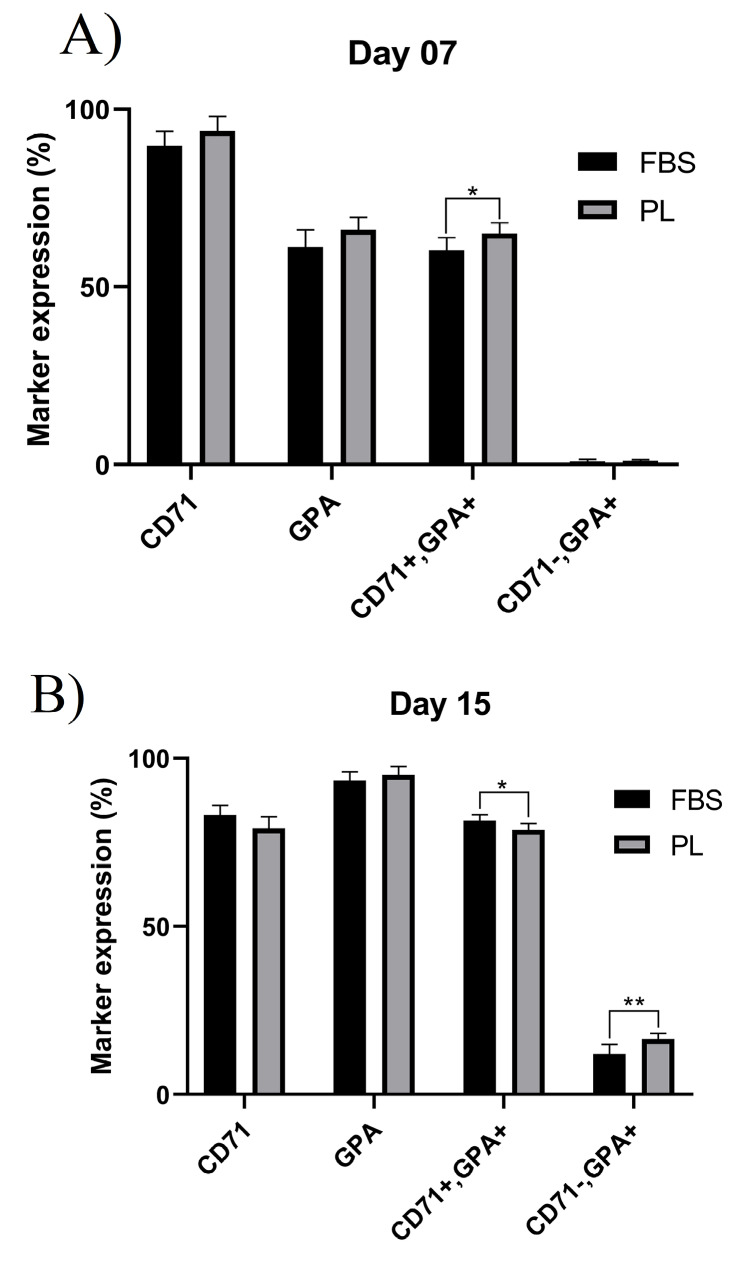
Flow cytometry analysis of erythroid cells surface markers cultivated in PL or FBS-containing media.(**A)** Percentage of erythroid surface markers at day 7. (**B)** Percentage of erythroid surface markers at day 15.

Figures 3B and C show the glycophorin A (GPA, CD235a) and transferrin receptor (CD71) markers on days 0, 7, and 15. CD71 expression levels were increased at the early stages of culture in both groups and decreased as the cells matured, while GPA expression level increased following maturation of cells; however, the difference between the expressions of these two markers was not statistically significant (*P* < 00.05) (Figures 5A and B). CD71-/GPA^+^ expression in day 15 cells was significantly higher in PL-containing medium compared to FBS-containing medium. The surface marker expression of cells at day 7 and 15 is shown in Table 2.

**Table 2:** Surface marker expression of differentiated erythroid cells at day 7 and 15 in medium supplemented with FBS or PL

**Marker**	**Day 7**			**Day 15**		
	**FBS**	**PL**	***P*** ** value**	**FBS**	**PL**	***P*** ** value**
CD71^+^	89.75 ± 4.09	93.96 ± 4.11	0.1059	83.06 ± 2.50	79.65 ± 3.50	0.0638
GPA^+^	61.29 ± 4.87	66.17 ± 3.56	0.0796	93.63 ± 2.64	95.11 ± 3.04	0.2844
CD71^+^/GPA^+^	60.32 ± 3.55	65.01 ± 3.04	0.0345	60.32 ± 1.74	87.31 ± 1.91	0.0253
CD71-/GPA^+^	0.97 ± 0.60	1.16 ± 0.34	0.5226	11.94 ± 2.91	16.4 ± 1.65	0.0084

Data are presented as mean± standard deviation of six independent experiments. FBS: fetal bovine serum; PL: platelet lysate; GPA: glycophorin A.

### Erythroid gene expression

**Figure 6:**
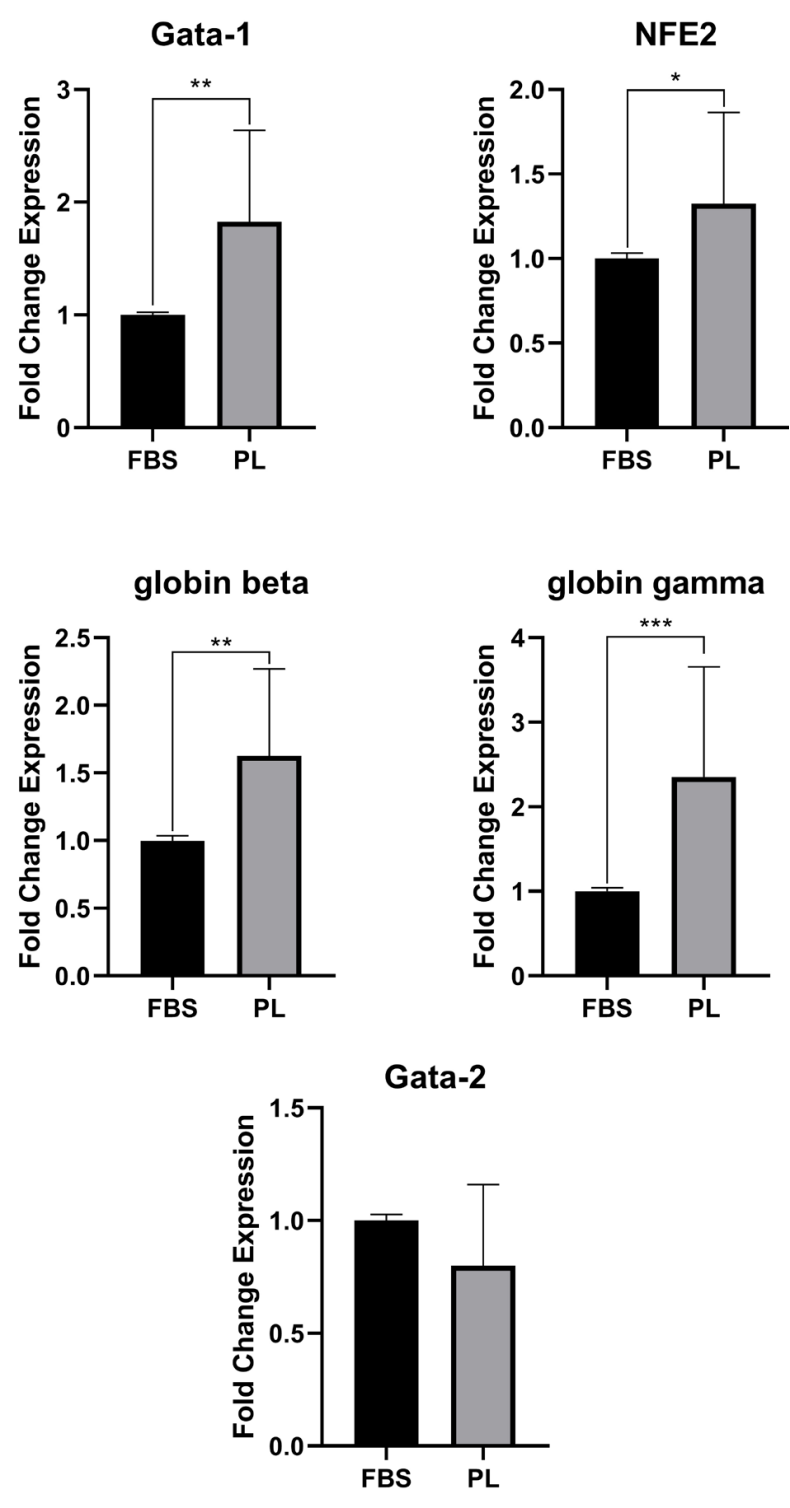
Gene expression analysis. The erythroid cells were harvested after 8 days from FBS-containing (black bars) or PL-containing (gray bars) media. Data are presented as mean± standard deviation from six independent experiments (*P < 00.05, **P < 00.01, ***P < 00.001).

Figure 6 shows the effect of PL on the expressions of erythroid differentiation genes. The expression of *GATA-1*, *NFE2*, g and b globins was significantly increased in cells cultivated in PL-containing medium compared to FBS-containing medium at day 8 (*P* < 00.05). However, *GATA-2* expression was decreased but was not significant among cells cultivated in PL-containing medium.

## Discussion

In this study, we for the first time investigated the effect of PL replacement on erythroid differentiation pattern and RBCs generation in humanized culture medium.

PL contains several growth factors and chemokines such as EGF, PDGF, VEGF, IGF, TGF-β, FGF, HGF, GM-CSF and C­XCL12, which make it a viable alternative for FBS. HSCs have receptors for these growth factors, which can increase erythroid cell proliferation and differentiation along with other cytokines.^11,12^

In this research, the growth factors contained in PL had a higher concentration than that of FBS growth factors measured by other researchers.^13,14^ This feature may eliminate the need for adding growth factors during cell culture to increase cell proliferation and differentiation. In several studies, PL growth factors have been measured, in some of which the growth factors were close to our study^13,15,16^ and were higher in others.^17-19^ In this study, the concentration of growth factors present in PL was measured in 5 platelet bags combined to investigate the extent of its variation in different samples, which standard deviation in each sample shows these changes. These finding may be related to the effect of platelet count, platelet source, preparation method, storage and difference of each PL sample with another. In other words, PL may have batch-to batch variation, which can be mitigated by pooling more platelet bags.

The effect of PL has also been evaluated in numerous studies on various cells such as the differentiation of MSCs and dendritic cell from monocytes. The effect of PL on the cells has been considerable and it has even been suggested as an alternative for FBS.^20-23^ In our study, PL significantly increased MSCs expansion compared to FBS-containing medium. However, Abdelrazik et al^24^ study showed that PL was able to attenuate the inhibitory effect of MSCs on T-cells and NK cells, thereby reducing the immunomodulatory effect of MSCs. Different concentrations of growth factors in PL than FBS reason for the change in cell conditions.

A single unit of blood with an acceptable RBC count for injection contains approximately 2×10^12^ RBCs.^10^ RBC counts could be increased through culture and differentiation of HSCs into erythroid cells.

In our study, the maximum rate of cell expansion was observed at day 15 in PL-containing medium compared to FBS-containing one. Given that each cord blood unit contains 2-4×10^6^ CD34^+^ cells, at this rate of cell expansion (99×10^3^ fold), RBC count of each cord blood unit can reach the number of RBCs with 120-day lifespan for all erythrocytes, which are injectable to the patients needing repeated blood transfusions to prevent iron overload or immune reactivity instead of RBCs donation with a 28-day lifespan.

Erythroid cells are derived from a progenitor called the common erythroid-megakaryocytic progenitor. The erythropoiesis process is characterized by specific markers such as CD71, GPA, band 3, and spectrin, which are specific components of the erythrocyte membrane.^6,25,26^

In this study, we investigated CD71 and GPA, known as erythroid differentiation markers, as well as early stage of reticulocyte.^27,28^ The expressions of CD71 and GPA were higher in cells cultivated in PL-containing medium than FBS-containing medium. In addition, on day 15, CD71- GPA^+^ expression was higher in cells cultivated in PL-containing medium than cells cultivated in FBS-containing medium, indicating higher erythroid differentiation and maturation rate in PL-containing medium.

Numerous studies have also evaluated the HSCs differentiation into erythroid cells,^1,6,8,10,11,28-31^ some of which investigated differentiated cells in vivo.^8,10,31^ These studies have employed different culture methods, cytokine cocktails and cells and have achieved various results. These findings indicate the effect of culture methods, the amount and type of growth factors and cytokines, the source of HSCs, and the effect of co-culture with feeder layer on erythroid cells differentiation. Examples of these effects include the impact of the cytokine cocktail, in which TPO and FL cytokines are suitable for HSCs survival and self-renewal; however, these cytokine are known to induce multi-lineage differentiation and reduce erythroid differentiation of HSCs.^28^ Additionally, MSCs and macrophages contribute to the final stages of erythroid differentiation and help enucleate these cells.^28^ MSCs can be isolated from several sources such as BM, adipose tissue, umbilical cord, umbilical cord blood, Wharton’s jelly, ammonia fluid, cervical tissue placentae, skeletal muscle tissue, dental pulp, and dermal tissues.^7^ Among the different sources of MSCs, BM-MSCs are considered as an appropriate source with the best characterized properties.^28^ Nonetheless, further studies are still needed to investigate the effect of different feeder layers on erythroid differentiation and maturation. Besides, research has shown that umbilical cord HSCs are appropriate sources for erythroid differentiation with high expansion rates.

## Conclusion

Several applications of RBCs differentiated *in vitro* have prompted us to humanize culture mediumfor erythroid differentiation of CD34^+^ HSC to generate RBCs *in vitro* more safely with a lower cost.

Our findings showed that PL with different growth factors could be a suitable alternative for FBS in HSCs culture and RBCs generation. However, PL replacement requires further study and examination of differentiated cells by in vivo studies. In addition, similar to FBS, PL has batch-to-batch variation and the method of PL preparation needs standardization and development of a standard protocol.

## Ethical Issues

Ethical approval was granted by Ethics Committee of Tabriz University of Medical Sciences (Ethics No. IR.TBZMED.REC.1396.1181). Cord blood was collected at Al-Zahra Hospital affiliated to Tabriz University of Medical Sciences from full-term healthy deliveries after obtaining informed consent from mothers.

## Conflict of Interest

Authors declare no conflict of interests.

## Acknowledgments

This work has been done as part of an MSc thesis by Majid Zamani. This study was supported by Immunology Research Center at Tabriz University of Medical Sciences, Iran (grant No. 58826). The authors would like to acknowledge Immunology Research Center and Department of Immunology at Tabriz University of Medical Sciences (Iran) for their contribution.
